# Impact of B and P Doping on the Elastic Properties of Si Nanowires

**DOI:** 10.3390/nano15030191

**Published:** 2025-01-25

**Authors:** Nedhal Ali Mahmood Al-Nuaimi, Angela Thränhardt, Sibylle Gemming

**Affiliations:** 1 Theoretical Physics, Institute of Physics, Chemnitz University of Technology, D-09107 Chemnitz, Germany; angela.thraenhardt@physik.tu-chemnitz.de (A.T.); sibylle.gemming@physik.tu-chemnitz.de (S.G.); 2 Center for Materials, Architectures and Integration of Nanomembranes, Chemnitz University of Technology, D-09107 Chemnitz, Germany

**Keywords:** Si nanowire, doping, ABINIT, simulation, mechanical properties

## Abstract

Using gradient-corrected density functional theory we investigate the mechanical properties of ultrathin boron (B) and phosphorus (P) doped silicon nanowires (SiNWs) along the [001] and [111] orientations within the PBE approximation. Both pristine and doped SiNWs under study have diameters ranging from 5 to 8 Å. Our results show that doping significantly enhances the bulk modulus ($B_{0}$), shear modulus ($G_{V}$), Young’s modulus (*Y*), and other mechanical parameters. The significant anisotropy observed in the mechanical properties of Si[111] NWs, with varying moduli along different axes, further illustrates the complex interplay between mechanical behavior and electronic structure at the nanoscale. The mechanical flexibility of SiNWs, combined with their tunable electronic properties due to quantum confinement, makes them promising candidates for advanced nanoelectronic devices, nanoelectromechanical systems (NEMS), and advanced technologies.

## 1. Introduction

Silicon, atomic number 14, plays a vital role in the electronics, construction, and renewable energy sectors [1,2]. Its properties as a semiconductor integrate it into devices like computers and smartphones [3,4,5,6]. Silicon’s role in photovoltaic cells underscores its importance in sustainable technology [7,8], demonstrating its versatility across industries.

Transitioning from bulk silicon (Si) to silicon nanowires (SiNWs) enhances the scope of silicon’s applications due to their high surface-to-volume ratio and quantum confinement effects [9]. SiNWs exhibit superior electronic and optical properties, making them suitable for nanoelectronics, sensors, and energy storage [10,11,12,13]. They are particularly promising for high-performance lithium-ion batteries [14,15] and flexible electronics [16,17,18], marking a significant advancement in material science [19,20,21].

SiNWs require elaborate synthesis and handling, with precise temperature control and chemical purity being crucial [10,13]. Bottom-up growth, top-down fabrication and mixed routes to SiNWs have been reported [22,23] including direct doping schemes e.g., by ion beam implantation [24]. Theoretical studies, supported by computational simulations, guide experimental research by predicting SiNW behavior under various conditions, saving resources [11,25,26,27]. Understanding the anisotropic elastic properties of SiNWs, particularly those oriented along the [001] and [111] directions, is crucial for their integration into nanoelectromechanical systems (NEMS) and flexible electronics [28,29,30], as these properties directly influence the design of devices leveraging the unique mechanical characteristics of SiNWs.

In general, the 4th-rank tensor of the elastic constants $c_{ijkl}$ relates the 2nd-rank tensors of strain $\varepsilon_{kl}$ and stress $\sigma_{ij}$ in a linear fashion as $\sigma_{ij}=\sum_{kl}c_{ijkl}\varepsilon_{kl}$ [31,32]. The tensor $c_{ijkl}$ in its most general form has 3 × 3 × 3 × 3 = 81 components, which are the independent elastic constants.

For bulk silicon as reference compound for the nanowires the Voigt symmetry of the cubic diamond-type crystal structure requires that the constants $c_{ijkl}$ are symmetric with respect to the interchanges of the indexes (i,j), (k,l), and (ij,kl). The resulting forms of $\sigma_{ij}$ and $\varepsilon_{kl}$ are symmetrical and each contains six independent components. Under those conditions, only up to 36 independent elastic constants may be obtained [33] to link strain and stress states, which can be represented by the rank-two tensor of the stiffness matrix *C_ij_* and reads as follows:

(1) $$C_{ij}=\left[\begin{array}{cccccc} C_{11} & C_{12} & C_{13} & C_{14} & C_{15} & C_{16}\\ C_{21} & C_{22} & C_{23} & C_{24} & C_{25} & C_{26}\\ C_{31} & C_{32} & C_{33} & C_{34} & C_{35} & C_{36}\\ C_{41} & C_{42} & C_{43} & C_{44} & C_{45} & C_{46}\\ C_{51} & C_{52} & C_{53} & C_{54} & C_{55} & C_{56}\\ C_{61} & C_{62} & C_{63} & C_{64} & C_{65} & C_{66} \end{array}\right]$$

This matrix provides a comprehensive description of all linear elastic properties and interactions within the crystal structure. It is the starting point for the full derivation of the elastic properties of cubic materials. Exploiting the further symmetries of the diamond lattice reduces the number of independent components further, such that only the uniaxial *C*_11_, biaxial *C*_12_ and shear component *C*_44_ are needed to characterize the elastic properties of bulk silicon. Table 1 gives an overview of those elastic constants for bulk silicon obtained by the most prominent theoretical and experimental methods that have been employed in the study of silicon nanowires.

The overall trend observed in the table is that different computational methods lead to varying estimates of the elastic stiffness constants for silicon, whereas the experimental results agree within ±1% regardless of the employed measurement technique. The overbinding, which has been documented well for the Local Density Approximation (LDA) in DFT calculations [47] generally produces higher values for $C_{11}$, $C_{12}$, and $C_{44}$ compared to DFT within the Generalized Gradient Approximation (GGA). However, for bulk silicon the stiffness constants obtained from such first-principles calculations agree quite well with experimentally measured ones. On the other hand, simulations with the classical atomistic Stillinger-Weber potential (SW), which is an empirical method optimized to reproduce the melting point of bulk silicon, tends to give lower $C_{11}$ values but significantly higher values for $C_{12}$ and $C_{44}$, suggesting that it overestimates the material’s response to shear stress. In contrast, the macroscopic PK2 stress method, which models material deformation under large stresses using continuum elastics, consistently results in stiffness constants, which are even lower than the ones calculated with the DFT-GGA, indicating a softer material response. Within the simulation results, the GGA data provide a balanced estimate of the stiffness constants, with values generally falling between those obtained from first-principles calculations with the LDA and with classical empirical methods both in the atomistic picture given by the SW potential and in the continuum one with the PK2 stress approach.

The differences between experimental and theoretical values of the elastic stiffness constants arise primarily due to the contrasting nature of the methods employed. Theoretical models, such as those based on the DFT or empirical potentials, typically assume idealized, defect-free crystal structures and conditions such as zero temperature, leading to predictions that may not fully capture real-world complexities. Theoretical methods often provide an accurate representation of intrinsic material properties under perfect conditions but may overlook factors like defects, impurities, and temperature effects, which can significantly influence the material’s behavior. In contrast, experimental measurements may be affected by sample imperfections, environmental factors (e.g., temperature, pressure), and measurement uncertainties, which can lead to deviations from theoretical predictions. Furthermore, experimental techniques such as X-ray diffraction or ultrasonic measurements are subject to precision limitations, and the materials used in experiments may differ from those assumed in theoretical models.

As a result, while theoretical predictions provide valuable insights into the idealized properties of materials, experimental results reflect the material’s behavior under practical conditions, leading to inevitable discrepancies between the two. Among the simulation methods the differences in the results highlight the varying degrees of accuracy and assumptions underlying each computational method. Although DFT is the only theoretical approach, which is free of element-specific parameters, the results suggest that quantum mechanical first-principles calculations are best suited to determine the elastic properties of silicon-based structures, hence we employ it for the present study of SiNWs, which exhibit quantum confinement in two dimensions.

Our approach is supported by prior simulation studies on SiNWs in comparison with the corresponding experimental results: For [110] and [111] SiNWs DFT investigations showed that quantum size effects significantly modify the electronic properties below 0.7 nm diameter [48,49], whereas the elastic properties are still affected for diameters of 0.7 to 2.6 nm [34]. There, the compressive strain in [110] SiNWs significantly decreases deformation potentials, potentially enhancing electron mobility despite an increased effective charge carrier mass. In contrast, [111] SiNWs show minimal changes of the electronic properties under strain, while the strain can dramatically boosts electron and hole mobilities up to 100 times for hole mobility in [111] SiNWs [34]. Regarding the elastic properties of pristine SiNWs, DFT studies indicate a decrease of the Youngś modulus with decreasing diameter [50].

Plastic deformations including dislocations and stacking faults become relevant only for thicker wires, i.e., in the realm of classical atomistic modeling as highlighted by a study on the relation between the shape and mechanical properties of SiNWs [51]. There circular SiNWs exhibited a higher tensile yield stress of about 11.21 GPa, while square SiNWs demonstrated a greater compressive yield stress of around 8.89 GPa. Under tensile loading along the [110] direction, deformation primarily occured through slip on the [111] planes, whereas under compressive loading, the square SiNWs deform via slip on different planes. Similar results were obtained with the same method also for SiNW diameters down to 0.8 nm at elevated temperature [52]. In contrast, for more artificial, non-faulted wires comparable simulations of tensile loading resulted in higher Young’s moduli [53]. These results signify that the dominant slip planes depend on the type and directionality of the applied stress. Also the facet type of the wire was found of relevance in atomistic MD studies, as nanowires with {110} facets were stronger under tension, while those with {100} facets were stronger under compression; the effects were more pronounced in smaller nanowires due to the higher surface-to-volume ratio [54].

Experimentally, Si [111] NWs with diameters ranging from 100 to 700 nm have been studied by atomic force microscopy (AFM) multipoint bending tests. The results indicated that the overall Young’s modulus of [111] oriented Si nanowires increases from approximately 100 GPa to around 160-180 GPa as the diameter decreases [55]. AFM-based indentation measurement by Sohn et al. [56] focused on the local elastic properties of silicon nanowires and found them independent of the diameters within the range ∼80–∼600 nm. Similar findings have been obtained for other Vapor-Liquid-Solid (VLS) grown SiNWs within this diameter range [25,57,58,59,60]. For thinner wires between 15 nm and 60 nm Transmission Electron Microscopy (TEM) by Han et al. [61] revealed large-strain plasticity in SiNWs under tensile stress, initiated by dislocation emission and accompanied by local amorphization in the thin-diameter limit. That indicates that structural defects are important for the deformation behaviour of thick, bulk-like nanowires, but less relevant for the ultrathin structures of the present study, where stronger deviations from the bulk properties prevail [62]. Summarizing the experimental perspective, fabrication methods, processing conditions, and the presence of defects (such as cracks), impurities (like water), surface contamination, coatings, and the applied techniques are further factors that significantly influence the mechanical properties of the nanowires [55,57,60,63,64,65,66,67,68] and there is an obvious lack of measurements for very thin structures, already for the pristine SiNW case.

Even less data is available for the effect of substitutional doping on the elastic properties of silicon, in particular of SiNW: Continuous stiffness measurements (CSM) and pulse-echo techniques revealed that Young’s modulus of phosphorus-doped Czochralski-grown bulk silicon is approximately 162 GPa and 167 GPa for heavy and light doping [69]. Earlier results reported in Ref. [43] for P-doped float-zone silicon showed a stronger temperature dependence of the elastic constants, particularly the shear components, upon doping.

Theoretical studies of P-doped [100] SiNWs with a semi-continuum model indicated that Young’s modulus significantly decreases with the reduction in the nanowire’s width and thickness and with rising temperature [70]. First-principles studies have, to the best of our knowledge, been restricted to doped silicon nanocrystals [71,72,73]. As the size of Si-NCs decreases, dopant atoms like B and P tend to migrate towards the surface, leading to significant alterations in the electronic structure and optical properties [71] and the local bonding motifs and electron distributions in the frontier orbitals deviate significantly from the ones of pristine nanoclusters [72], also upon co-doping [73].

Theoretical studies on doped SiNW [10,71,74] solely focused on the electronic properties, whereas the impact doping on the elastic properties has received limited attention, so far. We, thus, build on our previous study [48] by providing a comprehensive investigation of the elastic and mechanical properties, following our initial calculations of the electronic structure. The theoretical framework rigorously examines how doping with boron (B) and phosphorus (P) influences the elastic properties of pristine SiNWs oriented along the [001] and [111] directions by first-principles modeling.

## 2. Methodical Details

### 2.1. Elastic and Mechanical Properties

Further key quantities that characterize the mechanical behavior of materials can be derived from the elastic constants described above, and we will discuss the most common measures in the following:

The bulk modulus, $B_{0}$, is a measure of a material’s resistance to uniform compression and is calculated as:

(2) $$B_{0}=\frac{1}{3}\left(C_{11}+2C_{12}\right)\;.$$

The shear modulus $G_{\nu }$ reflects the material’s response to shear stress and is given by:

(3) $$G_{\nu }=\frac{1}{5}\left(3C_{44}+C_{11}-C_{12}\right)=\frac{1}{5}\left(3C_{44}+2C^{\prime }\right)\;.$$

Young’s modulus *Y* is a measure of the stiffness of a material and is defined as:

(4) $$Y=\frac{9G_{\nu }B_{0}}{3B_{0}+G_{\nu }}\;,$$

where $B_{0}$ and $G_{\nu }$ enter as functions of $C_{ij}$. Poisson’s ratio ν describes the ratio of transverse strain to axial strain under uniaxial stress. It reflects changes of the average density, which occur upon uniaxial deformation:

(5) $$\nu =-1+\frac{Y}{2G_{\nu }}=\frac{3B_{0}-Y}{6B_{0}}=\frac{1}{2}-\frac{Y}{6B_{0}}\;.$$

The Lamé constants λ and μ are fundamental parameters in the linear elasticity theory. In a generalized formulation of Hooke’s law they describe the first and second invariant of the quadratic dependence of the potential on the strain required by the linear force-strain relationship:

(6) $$\lambda =\frac{Y\nu }{\left(1+\nu )(1-2\nu \right)}\;,$$

(7) $$\mu =G_{\nu }=\frac{Y}{2(1+\nu )}\;.$$

The Kleinman parameter ζ relates to internal strain relaxation by bond bending (ζ=0) and bond stretching (ζ=1) and is defined as:

(8) $$\zeta =\frac{C_{11}+8C_{12}}{7C_{11}+2C_{12}}\;.$$

The shear constant $C^{\prime }$ and Cauchy’s pressure $C^{\prime \prime }$ are given by:

(9) $$C^{\prime }=\frac{1}{2}\left(C_{11}-C_{12}\right)\;,$$

(10) $$C^{\prime \prime }=\left(C_{12}-C_{44}\right)\;,$$

where the Cauchy pressure indicates the propensity of a material towards ductile or brittle deformation behaviour by a positive or negative sign.

In SiNWs, for the lateral axis, the moduli are determined using $C_{11}$, $C_{12}$, and $C_{44}$, whereas for the longitudinal axis, the moduli are obtained by the symmetry of the system which requires utilizing $C_{33}$, $C_{13}$, and $C_{66}$. That is due to the relationships $C_{11}=C_{22}\neq C_{33}$, $C_{12}=C_{21}\neq C_{23}=C_{32}=C_{13}=C_{31}$, and $C_{44}=C_{55}\neq C_{66}$, which reflect the lower symmetry of the SiNW with respect to bulk silicon and the subsequent different responses to stress along the lateral and longitudinal directions.

These equations provide a fundamental understanding of the mechanical properties of SiNWs, which are critical for their application in various nanotechnological fields. The values of these parameters are influenced by factors such as crystallographic orientation and doping, which can be tailored to optimize the performance of SiNWs in specific applications [55,56,75,76,77].

### 2.2. Model Systems

All nanowire structures, i.e., pristine SiNWs oriented along the [001] and [111] directions and their B and P-doped analogs, were built using the software VESTA [78]. Initially, we started with a primitive unit cell of bulk silicon (Si) from the American Mineralogist database [79].

The optimized structure served as the building block for constructing well-defined SiNW supercells oriented along either the (001) or (111) plane. The nanowires were then constructed in the desired direction along the *z* axis, with periodic boundary conditions applied in all three spatial directions. To ensure the model structures represented truly one-dimensional SiNWs, a vacuum space of 15 Å was added in both the *x* and *y* directions, large enough to eliminate interactions between the nanowire and its periodic replicas in adjacent simulation cells.

The repeated structural unit contains 13 atoms for [001] SiNWs and 22 atoms for [111] SiNWs. For doping studies, one silicon atom was replaced with either a B or P atom, and symmetrically inequivalent positions were investigated separately.

In this investigation, the atomic positions of the bulk-derived starting structures for the pristine SiNWs were structurally relaxed according to forces obtained from first-principles, employing gradient-corrected density-functional calculations.

**Figure 1:**
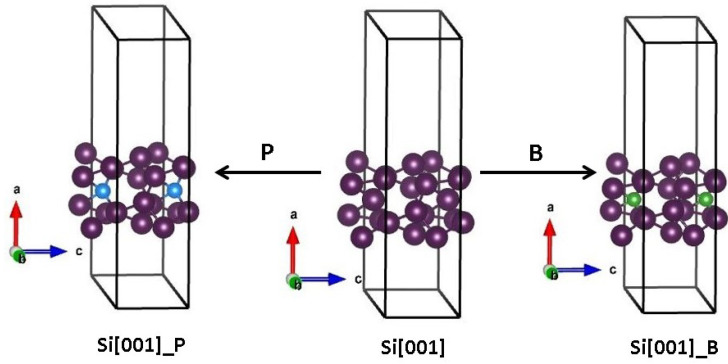
The atomic structures of: SiNWs[001] with P, SiNWs[001], and SiNWs[001] with B; Si atoms are shown in dark purple, B atoms in green, and P atoms in blue. The first replica of dopant atom is also shown.

**Figure 2:**
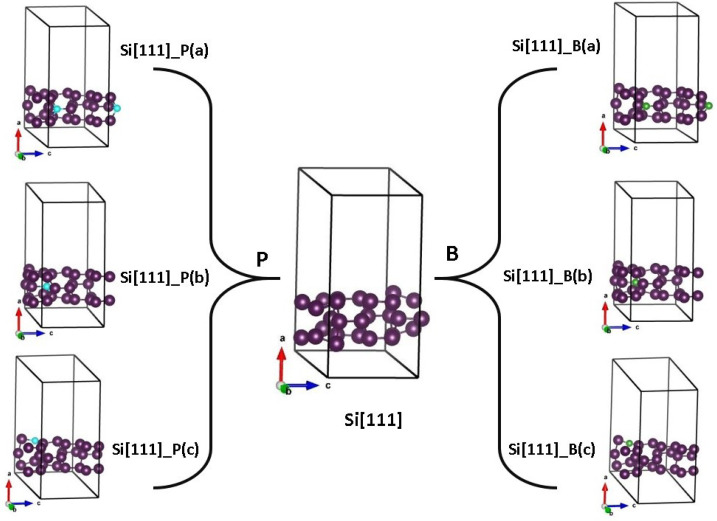
Atomic structure of SiNWs[111], SiNWs[111] doped with boron (**right panel**), and SiNWs[111] doped with phosphorus (**left panel**) at positions a, b, and c. Silicon atoms are depicted in dark purple, boron atoms in green, and phosphorus atoms in blue. The labels a, b, and c represent the substitution of dopants at three distinct positions within the SiNWs[111] structures. The first replica of dopant atom is also shown.

These relaxed pristine structures served as starting points for further optimization of B- and P-doped SiNWs. The resulting atom configurations, representing stable minima of the total energy hypersurface, maintain the same local coordination of constituent atoms along the wire direction as the starting structures. The resulting structures are depicted in Figure 1 for the [001] direction and in Figure 2 for [111].

### 2.3. Computational Details

We conducted all electronic structure calculations using density functional theory (DFT) and density functional perturbation theory (DFPT) as implemented in the ABINIT code. The generalized gradient approximation (GGA) with the Perdew–Burke–Ernzerhof (PBE) parameterization was employed, and the core-valence interactions were described using the norm-conserving pseudo-potential method. An energy cutoff value of 20 Ha was applied for optimizing bulk silicon and 12.25 Ha for all nanowires. This choice was based on convergence tests that confirmed the adequacy of these values for accurate and efficient optimization of bulk silicon and nanowires [80,81].

In the self-consistent potential and total energy calculations, the Brillouin zone was sampled in *k* space within the Monkhorst-Pack scheme using (1 × 1 × 16) for SiNWs and (6 × 6 × 6) for bulk silicon. The convergence criterion for total energy was set to 10^−6^ Ha per atom. All atomic positions and lattice parameters were optimized until the absolute value of the force acting on each atom was less than 5 × 10^−6^ eV/Å, ensuring minimization of total energy and atomic forces. The elastic constant tensor (*C_ij_*) was obtained using the linear-response method in the ABINIT code.

The mechanical behavior of SiNWs is characterized by various parameters such as the bulk modulus ($B_{0}$), shear modulus ($G_{\nu }$), Young’s modulus (*Y*), Poisson’s ratio (ν), and Lamé constants (λ and μ). These parameters are derived from the stiffness coefficients of the material, represented by the elastic stiffness matrix. In the following, we present the relevant equations that describe these properties.

## 3. Results and Discussion

The elastic stiffness constants $C_{11}$, $C_{12}$, and $C_{44}$ of silicon are the fundamental parameters that characterize its mechanical behavior under stress. We obtain the following reference values for these constants for bulk silicon: $C_{11}=152.53$ GPa, $C_{12}=56.21$ GPa, and $C_{44}=74.76$ GPa. A comparison with the data from previous studies reveals very good agreement with very similar earlier DFT calculations with the GGA functional. Hence our numerical settings are adequate and the slight underestimation of the experimentally measured elastic properties in the GGA is reproduced for the silicon bulk. The close agreement between theoretical and experimental values underscores the reliability of first-principles approaches in predicting at least trends for the elastic properties, which are crucial for applications in semiconductor technology and structural materials science.

Let us inspect Table 2 in more detail to provide a comprehensive analysis of the mechanical properties of bulk silicon compared to the published experimental and calculated values.

The bulk modulus ($B_{0}$) obtained in our work is 88.32 GPa, which is lower than the experimental value of 98 GPa and several calculated values, such as 100 GPa [82], 92 GPa [83], and 100.7 GPa [40]. This suggests that our method underestimates the resistance of bulk silicon to uniform compression compared to other studies. This discrepancy could be attributed to the different exchange-correlation functionals employed in the two studies, specifically the use of LDA and GGA. However, the shear modulus ($G_{V}$) in our study is found to be 64.12 GPa. This value is higher than the experimental measurement of 52 GPa [84] and indicates that our approach overestimates the material’s rigidity.

For Young’s modulus (*Y*), our work gives a value of 154.88 GPa, which is lower than the experimental value of 185 GPa [84] and the calculated value of 168.5 GPa [40]. This discrepancy implies that our calculations underestimate the tensile stiffness of silicon, which might affect predictions of mechanical behavior under tensile loads.

The Poisson’s ratio (ν) calculated in our study is 0.207, which is lower than both the experimental value of 0.28 [84] and the calculated value of 0.247 [40]. This lower value suggests that our silicon model might predict less lateral expansion under compressive axial stress than observed or predicted by other studies, potentially affecting deformation predictions.

The Lamé constants (λ and μ) also show discrepancies. The value of λ in our work is 45.56 GPa, compared to the experimental value of 64 GPa [85] and the calculated value of 66.02 GPa [40]. The value of μ is 64.12 GPa, while the experimental value is 79.6 GPa [85] and the calculated value is 67.55 GPa [40]. The lower value of the Lamé constants in our work suggests that our study may predict the material as less stiff than what is generally measured, potentially affecting the predictions of the mechanical response under various loading conditions.

The Kleinman parameter (ζ) from our study is 0.51, which is close to the experimental and calculated values of 0.54 [86,87,88]. This signifies that our estimation of the bond bending and stretching forces is relatively accurate, suggesting that the first-principles method describes the experimental bonding character very well.

The shear constant ($C^{\prime }$) and Cauchy’s pressure ($C^{\prime \prime }$) values are in good agreement with the other first-principles results [38,39]. Our calculated shear constant of $C^{\prime }=48.1$ GPa falls between the values of 45.5 GPa in [38] and 50.6 GPa in [39]. Similarly, the calculated Cauchy pressure of $C^{\prime \prime }=-18.55$ GPa aligns well with the values of −17.7 GPa and −21 GPa reported in [38,39], respectively. These comparisons validate the reliability of our approach in capturing the mechanical properties of silicon, particularly its covalent nature as reflected in the negative $C^{\prime \prime }$ values.

In our study, the bulk modulus, Young’s modulus, Poisson’s ratio, and the Lamè constants are lower than those reported in the literature, in particular with classical methods, which are fitted including experimental values. This may indicate an underestimation of the material’s stiffness, yet still lies within the accuracy window discussed in the round-robin comparison of different DFT implementations in ref. [47]. The Kleinman parameter aligns very well with published values., thus, the overall agreement highlights the robustness and accuracy of our calculations [77].

**Table 2:** Bulk modulus ($B_{0}$), shear modulus ($G_{V}$), Young’s modulus (*Y*), Poisson’s ratio (ν), Lamé constants (λ), Lamé constants (μ), Kleinman parameter (ξ), Shear constant ($C^{\prime }$) and Cauchy’s pressure ($C^{\prime \prime }$) for bulk silicon compared with those of experimental and calculated values.

	Bulk Si		
	Our Work	Exp.	Cal.
Bulk modulus $B_{0}$ (GPa)	88.32	98 [82,89]	100 [82], 92 [83], 100.7 [40]
shear modulus $G_{V}$ (GPa)	64.12	52 [84]	67.55 [40]
Young’s modulus *Y* (GPa)	154.88	185 [84]	168.5 [40]
Poisson’s ratio ν (GPa)	0.207	0.28 [84]	0.247 [40]
Lamé constants λ (GPa)	45.56	64 [85]	66.02 [40]
Lamé constants μ (GPa)	64.12	79.6 [85]	67.55 [40]
Kleinman parameter ξ (GPa)	0.51	0.54 [86]	0.54 [87,88]
Shear constant $C^{\prime }$ (GPa)	48.1		45.5 [38], 50.6 [39]
Cauchy’s pressure $C^{\prime \prime }$ (GPa)	−18.55		−21 [38], −17.7 [39]

Our previous study of the electronic structure of doped Si and SiNWs provides a fundamental understanding that can help explain the results we have obtained here for the elastic properties [48]. Pristine bulk silicon (Si) is known for its robust mechanical properties, which include a high bulk modulus of 88.32 GPa, a shear modulus of 64.12 GPa, and a Young’s modulus of 154.88 GPa. These values indicate that bulk Si has high resistance to deformation both under uniform pressure and under shear stress. The mechanical stability of bulk Si is complemented by its electronic properties, particularly its indirect band gap of 1.12 eV [48]. The combination of high mechanical strength and suitable electronic properties makes bulk Si an ideal material for various electronic and structural applications, particularly in semiconductor technology.

The calculated Poisson’s ratio of 0.207 indicates moderate lateral compression under tensile stress along the wire, a characteristic typically observed in materials with good ductility and toughness. This value reflects the elastic behavior predicted by our computational model for bulk silicon. These mechanical properties ensure that defect free bulk Si can sustain substantial loads and resist fractures, which is critical for maintaining the integrity of semiconductor devices under operational stresses [90,91].

The mechanical properties of NWs vary with the crystallographic orientation along both lateral and longitudinal axes. Thus, both lateral and longitudinal analyses are essential for optimizing SiNWs in applications e.g., flexible electronics, where directional mechanical properties are important [20,28,29,30].

In the present discussion, we follow the procedure outlined in Ref. [92] to address the anisotropy of the system. Our calculations provide elastic tensor data for both relaxed and clamped ion configurations. In the relaxed ion case, both the lattice and ions adjust to applied stress, offering a realistic view of material behavior under conditions like thermal expansion. The ions remain fixed in the clamped ion scenario, isolating the elastic response from one of the lattice alone. Comparing these results helps distinguish between pure lattice effects and combined lattice-ionic contributions, offering deeper insight into the material’s mechanical properties. For ideally periodic silicon, the off-diagonal terms $C_{12}$, $C_{21}$, $C_{13}$, and $C_{31}$ are equivalent for symmetry reasons. However, in nanowires, this symmetry is broken, Thus, the elastic constants $C_{11}$, $C_{12}$, and $C_{44}$ specifically govern the calculation of the lateral modulus, which describes the response of the material to deformation along directions perpendicular to the nanowire axis. The lateral modulus provides important information about the stiffness of the material in these directions and is essential for applications where lateral mechanical stresses are relevant, such as NEMS. In contrast, the longitudinal modulus, which describes the material’s response to deformation along the axis of the nanowire, is calculated using the constants $C_{13}$, $C_{33}$, and $C_{66}$. These constants are especially important for understanding the mechanical behavior of nanowires subjected to tensile or compressive loads along their length.

The data presented in Table 3 and Table 4 compare the elastic tensor values for relaxed and clamped ion configurations, respectively, for Si[001], Si[001]-B, and Si[001]-P NWs. Generally, the elastic constants are higher in the clamped ion case, reflecting a stiffer response. For instance, $C_{11}$ for Si[001]-B increases from 0.0687 (relaxed) to 0.0953 (clamped), and similar trends are observed for other constants like $C_{44}$. This highlights the softening of the wires due to ionic relaxation, whereas lattice relaxation alone, with clamped ions, results in greater stiffness.

The presented Table 5 provides a comprehensive comparison of several mechanical properties for both relaxed and clamped ion configurations of Si[001] NWs in both lateral and longitudinal axes. A key observation is the distinct difference between the relaxed and clamped ion cases. For instance, the bulk modulus $B_{0}$ is reduced from 1.21 GPa (clamped) to −0.5 GPa in the fully relaxed case, due to the strong anisotropy of the wire. Upon full relaxation, the coordination of the Si atoms within the wire deviates significantly from the original crystal structure. Similarly, the Young’s modulus, which measures the stiffness of a material under tension, shows a significant decrease in both the lateral (from 2.96 GPa to −0.928 GPa) and longitudinal directions (from 3.83 GPa to 1.448 GPa) upon full relaxation.

This underpins the analysis of the bulk modulus: the periodicity along the wire direction ensures that Young’s modulus is smaller than that of bulk Si and remains positive for both clamped and relaxed structures. Perpendicular to the wire, however, Young’s modulus becomes negative once the regular ionic arrangement of the bulk structure is relaxed, due to the finite size effects described in [48,49,93,94]. In the clamped configuration, the structural sorting effect driven by ionic relaxation becomes evident, highlighting the impossibility of suppressing ionic relaxation in experimental conditions. We should be aware that clamping ions is not feasible in reality, as ionic relaxation naturally occurs to minimize the system’s energy. While computational models can artificially “clamp” ions by fixing their positions to study specific effects, this approach is not achievable in real-world experiments.

The shear modulus ($G_{V}$) and Lamé constant (μ) also follow a similar trend, indicating reduced resistance to shear deformation in the fully relaxed case. Moreover, the Kleinman parameter (ξ) and Cauchy’s pressure ($C^{\prime \prime }$) show contrasting behavior between the two configurations. For example, $C^{\prime \prime }$ becomes negative (−1.77 GPa) in the laterally clamped case, suggesting a significant shift in internal force balance when the ions are immobilized. These shifts in elastic properties underscore the considerable influence of ionic relaxation on the mechanical behavior of Si[001] NWs.

Negative values of mechanical properties have also been reported by Lee and Rudd [50] for Si[001] NWs, indicating unusual and unstable behaviors, in contrast to the stable characteristics of bulk materials. Factors contributing to these anomalies include high surface-to-volume ratio inducing compressive surface stresses [95], quantum confinement effects altering electronic structure and bonding [96], defects and impurities creating localized stress fields [97], and potential inaccuracies in experimental measurements or computational models [98]. Moreover, the bulk modulus can become negative prior to the phase transition as reported by Levanyuk et al. [99] for BaTiO_3_ and PbTiO_3_ films on a (001) cubic substrate.

In this study, we first focus on the elastic constants derived from the clamped-ion configuration. This clamped ion model serves as an intermediate approach to isolate the crystal lattice response and differentiate it from ionic relaxations. In this way, the influence of finite size effects can be separated from the effects of p-type and n-type doping.

Table 6 summarizes a comprehensive overview of the elastic constants (clamped ion) $C_{11}$, $C_{12}$, $C_{13}$, $C_{33}$, $C_{44}$, and $C_{66}$ for Si[111] NWs, along with their doped counterparts, Si[111]-P and Si[111]-B. The table is divided into three sections, corresponding to three different substitution positions for the dopants, labeled as a, b, and c see Figure 2.

In general, the elastic constants exhibit notable variations across the different configurations. For instance, with a value of 0.1572 the $C_{11}$ constant for pure Si[111] is significantly higher than the corresponding values for the doped NWs (P and B), which range from 0.1009 to 0.1086. This reduction in $C_{11}$ for the doped structures suggests that the introduction of dopants modifies the interatomic interactions, both due to electronic and size effects consequently impacting the material’s stiffness.

Furthermore, the off-diagonal elastic constants, such as $C_{12}$ and $C_{13}$, also show fluctuations in values, with $C_{12}$ = 0.0264 in Si[111], while the values for Si[111]-P vary from 0.0278 to 0.0318 across different positions. These differences emphasize how the structural and mechanical properties of silicon nanowires can be tuned through careful doping strategies. Additionally, $C_{44}$ and $C_{66}$ indicate a somewhat consistent trend, albeit with lower values compared to the bulk elastic constants. The $C_{44}$ values range from 0.0306 to 0.0404 for the doped (P and B) NWs, whereas $C_{66}$ exhibits a slight decrease with doping, indicating that the dopants may affect the shear resistance of the material.

The following section discusses the lateral and longitudinal mechanical properties in more detail, utilizing still the elastic constants of the clamped ion model structure.

Table 7 and Table 8 present the elastic properties for Si[001], Si[001]-B, and Si[001]-P nanowires (NWs) in the lateral and longitudinal axes, respectively. In Table 7, the elastic properties in the lateral direction (Table 7) show that the bulk modulus ($B_{0}$) for Si[001] is 1.21 GPa and increases significantly to 6 GPa for Si[001]-B, and slightly less to 5.68 GPa for Si[001]-P. This trend is mirrored in Young’s modulus (*Y*), where values of 2.96 GPa for Si[001] and 10.93 GPa for Si[001]-B indicate a substantial increase due to boron doping, while for Si[001]-P, it remains close to 10.85 GPa. And also in the longitudinal axis the Young’s modulus (*Y*) exhibits a marked increase upon doping (Table 8), with pure Si[001] NWs at 3.83 GPa, compared to 14.62 GPa for Si[001]-B and 13.27 GPa for Si[001]-P. Indicating that doping enhances the stiffness of SiNWs confirms a trend which has also been observed in other studies [100,101].

Table 8 highlights the elastic constants along the longitudinal axis, where the bulk modulus for Si[001] increases to 3.13 GPa and further rises to 9.9 GPa for Si[001]-B, and to 8.61 GPa for Si[001]-P. In contrast to Table 7, almost all of the values are positive.

The shear modulus ($G_{V}$) demonstrates a marked increase in both axes, with values for the longitudinal axis consistently higher than those for the lateral axis, particularly for Si[001]-B, which shows 5.83 GPa in the longitudinal axis compared to 4.572 GPa in the lateral axis. This reveals that the material exhibits enhanced stiffness and stability under longitudinal loading compared to lateral loading [102,103].

The elastic properties of bulk silicon, as detailed in Table 2, show notable differences when compared to the elastic properties of Si[001] nanowires presented in Table 7 and Table 8. Starting with the bulk modulus ($B_{0}$), bulk silicon exhibits a significantly higher value of 88.32 GPa, while the Si[001] nanowires show much lower values of 1.21 GPa in Table 7 (lat.) and 3.13 GPa in Table 8 (long.). This stark contrast suggests that bulk silicon is far more resistant to uniform compression than the nanowire structures.

In terms of the shear modulus ($G_{V}$), bulk silicon is reported at 64.12 GPa (around 67.55 GPa, Cal. [40] Table 2), which is significantly higher than the 1.356 GPa presented in Table 7 for the lateral direction and the 1.478 GPa shown in Table 8 for the longitudinal direction. The lower shear modulus in the nanowires suggests a reduced ability to withstand shear deformation, reinforcing the notion that nanostructures typically have lower stiffness than their bulk counterparts [9,40,82,84,89] and tend to bending deformations.

The Young’s modulus (*Y*) further illustrates this trend, with bulk silicon at 154.88 GPa (Exp. 185 GPa [84]) compared to 2.96 GPa in Table 7 and 3.83 GPa in Table 8 longitudinally.

Poisson’s ratio (ν) is another point of comparison, where bulk silicon has a value of 0.207 GPa. In contrast, the Si[001]-NWs have a Poisson ratio of 0.09 GPa laterally (Table 7) and 0.296 GPa longitudinally (Table 8). This variation suggests that the nanowires exhibit different lateral strain characteristics under axial loads compared to bulk silicon [85,86].

Examining Lamé constants, we find that bulk silicon’s λ is 45.56 GPa and μ is 64.12 GPa, whereas the values for NWs-Si[001] are significantly lower with 0.298 GPa and 1.357 GPa laterally and 2.14 GPa and 1.477 GPa longitudinally. This reduction further emphasizes the diminished elastic response of the nanowires compared to bulk silicon.

Finally, the Kleinman parameter (ξ) for bulk silicon is 0.51, while for the Si[001]-NWs, it is −0.1 perpendicular to the wire and 0.38 along the wire direction. This indicates that even along the periodic direction, the covalent character of the Si-Si bond is reduced. These findings are reminiscent of the polyamorphic transition in Ref. [104].

In the case of SiNW along the [111] direction, the mechanical properties demonstrate significant variations when doped with boron (B) or phosphorus (P), as shown in Table 9. These properties are measured along both lateral (lat.) and longitudinal (long.) axes.

The bulk modulus ($B_{0}$) for undoped Si[111] NWs is 7 GPa on the lateral axis and 8.68 GPa in the longitudinal axis. These values indicate a moderate level of stiffness, comparable to previously reported values for SiNW along the [111] direction, which range from 6.5 to 10 GPa for bottom-up VLS-grown [105] and top-down fabricated SiNW etched from a SiMox wafer with the BOX process [106]. When doped with B and P, the bulk modulus increases significantly in the longitudinal axis, with values as high as 9.74 GPa for Si[111]-P(c). This suggests enhanced stiffness due to doping, aligning with the literature indicating that doping can increase the mechanical strength of SiNWs [101].

The shear modulus ($G_{V}$) for undoped Si[111] NWs is approximately 5 GPa on both axes, reflecting the resistance of the material to shear deformation. Doping with B and P generally reduces the shear modulus in the lateral axis but increases it in the longitudinal axis, with the highest value of 5.04 GPa observed for Si[111]-B(c). This anisotropic behavior is consistent with prior studies showing that doping can significantly affect the shear properties of SiNWs depending on the doping element and concentration [107,108].

Young’s modulus (*Y*) for Si[111] NWs follows a similar trend, with undoped values of 12.11 GPa on the lateral axis and 12.5 GPa on the longitudinal axis. Aligned with our findings both Ref. [50] and Ref. [52] applied first-principles methods and demonstrated that the Young’s modulus of Si[111] NWs stays nearly consistent with the bulk value until the diameter drops below 6 nm.

The values of the Young’s modulus derived under certain assumptions are very low, between 2–20 GPa, representing 1–2 orders of magnitude below 170 GPa, the Young’s modulus of bulk Si along the (111) direction [109].

Doping with B and P results in a further decrease of Young’s modulus along the lateral axis but an increase the longitudinal axis, reaching a peak of 12.87 GPa for Si[111]-B(c) and 12.8 GPa for Si[111]-P(c).

Poisson’s ratio (ν) for undoped Si[111] NWs is 0.21 in the lateral axis and 0.26 in the longitudinal axis. This ratio generally increases with doping, particularly in the longitudinal axis, reaching up to 0.29 for B-doped (at a position) samples and 0.28 for P-doped (at c position) samples. These results demonstrate that doping significantly influences the mechanical behavior of SiNWs [70].

The Lamé constants also show significant anisotropy and doping dependence. For undoped Si[111] NWs, λ is 3.62 GPa laterally and 5.37 GPa longitudinally, while μ is around 5 GPa in both directions lat. and long.). Doping increases λ significantly for the longitudinal axis, up to 6.36 GPa for Si[111]-P(c), while μ remains relatively stable, highlighting the enhanced rigidity due to doping.

The Kleinman parameter (ξ) and shear constant ($C^{\prime }$) for Si[111] NWs show complex behavior with doping. The Kleinman parameter for undoped NWs is 0.32 laterally and 0.33 longitudinally. Doping generally increases ξ on the lateral axis but decreases it on the longitudinal axis, reflecting the discussed changes in bond flexibility [110]. The shear constant increases significantly along the longitudinal axis with doping, up to 10.07 GPa for Si[111]-P(c), indicating enhanced shear stiffness along this axis.

Finally, Cauchy’s pressure ($C^{\prime \prime }$) for undoped Si[111] NWs is −1.34 GPa laterally and 0.39 GPa longitudinally. Doping with B and P increases $C^{\prime \prime }$ on the longitudinal axis, indicating a transition toward more metallic bonding characteristics.

Doping Si[111] NWs with boron (B) and phosphorus (P) results in changed mechanical properties, which depend on the doping position (a, b, c). For instance, the bulk modulus for boron-doped Si[111] NWs ranges from 5.06 to 5.31 GPa (lateral) and 8.92 to 9.62 GPa (longitudinal), while for phosphorus-doped Si[111] NWs, it ranges from 5.45 to 5.53 GPa (lateral) and 9.07 to 9.74 GPa (longitudinal). The shear modulus also shows significant variability, indicating that mechanical properties are highly dependent on the specific doping configuration. These mechanical enhancements correlate with changes in the electronic band structure due to doping. Boron and phosphorus doping introduce acceptor and donor states, respectively, within the band gap of SiNWs, thereby altering their electrical properties. These modifications can enhance charge carrier mobility and improve conductivity, making doped Si[111] NWs highly suitable for high-performance electronic applications [48].

The elastic properties of Si[111] NWs (Table 9) exhibit significantly higher values compared to Si[001] NWs (Table 7 and Table 8). Si[111] NWs demonstrate a higher bulk modulus ($B_{0}$) of 7 GPa (lateral) and 8.68 GPa (longitudinal) compared to 1.21 GPa (lateral) and 3.13 GPa (longitudinal) for Si[001] NWs. The shear modulus ($G_{V}$) is also notably greater for Si[111] NWs, with values of 5 GPa (lateral) and 4.98 GPa (longitudinal), in contrast to 1.356 GPa (lateral) and 1.478 GPa (longitudinal) for Si[001] NWs. This reflects the fact that many Si-Si bonds are aligned parallel to the wire direction, while a larger component of the remaining bonds is oriented parallel to the lateral direction. All deformations necessitate significant changes in Si-Si bond lengths and Si-Si-Si bond angles, which are energetically unfavorable.

In line with this discussion, the Young’s modulus (*Y*) is much higher in Si[111] NWs, with values of 12.11 GPa (lateral) and 12.5 GPa (longitudinal), compared to 2.96 GPa (lateral) and 3.83 GPa (longitudinal) for Si[001] NWs. Poisson’s ratio (ν) is higher in Si[111] NWs, showing greater lateral strain with values of 0.21 (lateral) and 0.26 (longitudinal) versus 0.09 (lateral) and 0.296 (longitudinal) for Si[001] NWs.

In terms of the Lamé constants, Si[111] NWs display significantly larger values, indicating a stronger elastic response. The Kleinman parameter (ξ) values indicate that Si[111] nanowires exhibit a more favorable atomic displacement response under stress. This suggests that their atomic structure adjusts efficiently to deformation, likely due to the specific arrangement of bonds in the [111] orientation. Lastly, the shear constant ($C^{\prime }$) is notably higher in Si[111] NWs, indicating superior shear strength compared to Si[001] NWs.

When comparing the elastic properties of nano-wires Si[001] (Table 7 and Table 8) and Si[111] (Table 9) NWs with bulk silicon (Table 2), it is evident that both Si[001] and Si[111] nanowires exhibit significantly reduced mechanical properties relative to bulk silicon. The bulk modulus ($B_{0}$), shear modulus ($G_{V}$), and Young’s modulus (*Y*) in bulk silicon are much higher, indicating greater stiffness, with values like 88.32 GPa, 64.12 GPa, and 154.88 GPa, respectively. In contrast, Si[001] and Si[111] nanowires show markedly lower values for these properties, with the Si[111] NWs generally retaining slightly higher stiffness compared to Si[001] NWs. Moreover, Poisson’s ratio in nanowires is slightly lower than in bulk silicon, reflecting a higher tendency for deformation. Cauchy’s pressure ($C^{\prime \prime }$) is significantly less negative for nanowires, especially in Si[111] NWs, indicating an increased ductility compared to the more brittle bulk silicon. Overall, the reduction in dimensionality from bulk to nanowires leads to softer, more flexible structures, with Si[111] NWs retaining more of their bulk-like mechanical behavior compared to Si[001] NWs.

This substantial reduction in mechanical strength is mainly due to the lateral finite-size effects along with the high surface-to-volume ratio in nanowires, leading to increased surface area effects and decreased structural integrity [91,102,111,112,113,114].

In conclusion, nanowires exhibit significant reductions in elastic properties compared to bulk silicon, with Si[111] NWs generally retaining more of their original stiffness and strength than Si[001] NWs. However, both orientations are far softer and more flexible than bulk silicon, reflecting the impact of the reduced dimensionality on the mechanical behavior. A comprehensive analysis of pristine and doped SiNWs reveals distinct variations in mechanical properties influenced by crystal orientation and dopant type.

## 4. Conclusions

The mechanical properties of silicon nanowires (SiNWs) are significantly influenced by their crystallographic orientation and doping, as demonstrated by the comparison between Si[001] NWs and Si[111] NWs.

For Si[001]-B and Si[001]-P, doping significantly modifies the elastic stiffness constants ($C_{11}$, $C_{12}$, $C_{13}$, $C_{22}$, $C_{33}$, $C_{44}$, and $C_{66}$) and modulus values compared to pristine Si[001] NWs. Si[001]-B NWs exhibit increased bulk modulus ($B_{0}$) and Young’s modulus (*Y*), indicative of enhanced stiffness, whereas Si[001]-P NWs show higher shear modulus ($G_{V}$) and reduced Cauchy’s pressure ($C^{\prime \prime }$).

Si[001] NWs, particularly when doped, exhibit substantial improvements in mechanical properties compared to their undoped counterparts and in some cases surpass the mechanical enhancements seen in Si[111] NWs.

Similarly, for Si[111]-oriented NWs, mechanical properties vary across different dopant positions (a, b, c), with Si[111]-B NWs generally demonstrating superior elasticity relative to Si[111]-P NWs. However, the intrinsic mechanical properties of Si[111] NWs remain superior in their undoped state, emphasizing the directional dependence of SiNW mechanical properties. Our results align with literature reports and highlight the impact of doping on the mechanical properties of SiNWs, where doping significantly enhances stiffness and strength.

The enhanced mechanical properties due to doping suggest that the nanowires can maintain structural integrity under operational stresses, while the modified band structure enhances their electronic performance to suit specific application requirements.

This underscores the importance of crystallographic orientation and doping in customizing the mechanical properties of SiNWs. Future research should aim to refine theoretical models and experimental techniques to better predict and control the mechanical behavior of SiNWs, ensuring their optimal performance in advanced technological applications.

## Figures and Tables

**Table 1:** Values of the elastic stiffness constants *C*_11_, *C*_12_, and *C*_44_ of bulk silicon in units of GPa.

		*C*_11_ (GPa)	*C*_12_ (GPa)	*C*_44_ (GPa)
Leu et al. [34]	Cal. GGA	154.6	57.5	75.3
Zhu et al. [35]	Cal. SW pot	151.6	76.5	84.8
Beckstein et al. [36]	Cal. LDA	163.45	62.13	79.85
Wang et.al. [37]	Cal. LDA	161.86	63.58	77.53
Cao et al. [38]	Cal. PK2 stress	142	51	72
Pandit et.al. [39]	Cal. PK2 stress	161	64	76
Al-Douri et al. [40]	Cal. EPM	179	77.1	81.6
H. J. McSkimin [41,42]	Exp.	165.77	63.92	79.62
J. J. Hall [43]	Exp.	165.64	63.94	79.51
Cousins et.al. [44]	Exp.	167.54	64.92	80.24
D. F. Nelson [45]	Exp.	165	63	79.1
M. Shur [46]	Exp.	166	64	79.6

The local-density approximation (LDA), the Stillinger and Weber potential (SW pot), The second Piola-Kirchhoff stress tensor (PK2 stress), Empirical Pseudopotential Method (EPM), and generalised gradient approximation (GGA). Refs. [41,42] Ultrasonic Testing Technique. Ref. [43] Integrate Sound Velocity Measurement with theoretical predictions based on Keyes’s theory. Ref. [44] Energy-dispersive X-ray diffraction (EDXRD) and 006 Forbidden Reflection.

**Table 3:** Elastic tensor (relaxed ion) $C_{11}$, $C_{12}$, $C_{13}$, $C_{33}$, $C_{44}$, and $C_{66}$ in units (×10^2^) GPa, for Si[001] NWs, Si[001]-B NWs and Si[001]-P NWs.

	Si[001]	Si[001]-B	Si[001]-P
$C_{11}$	−0.0154121	0.0687089	0.3510459
$C_{12}$	0.0001213	0.0510547	0.3132239
$C_{13}$	−0.0030043	−0.0343987	0.0184246
$C_{33}$	0.0370765	0.0759791	0.0821149
$C_{44}$	−0.0013243	0.0537322	0.0090135
$C_{66}$	−0.0038042	0.1218119	0.0177484

**Table 4:** Elastic tensor (clamped ion) $C_{11}$, $C_{12}$, $C_{13}$, $C_{33}$, $C_{44}$, and $C_{66}$ in units (×10^2^) GPa, for Si[001] NWs, Si[001]-B NWs and Si[001]-P NWs.

	Si[001]	Si[001]-B	Si[001]-P
$C_{11}$	0.0255748	0.0952832	0.0911138
$C_{12}$	−0.0054358	0.0424793	0.0397254
$C_{13}$	0.0150906	0.0446189	0.0437713
$C_{33}$	0.0640266	0.2080383	0.1710644
$C_{44}$	0.0123334	0.0586393	0.0593981
$C_{66}$	0.0083058	0.0427187	0.0466322

**Table 5:** Elastic and mechanical constants in lateral axis (lat.) and longitudinal axis (long.) for both relaxed and clamped ion of Si[001] NWs.

	Si[001]-Relaxed Ion		Si[001]-Clamped Ion	
	Lat.	Long.	Lat.	Long.
Bulk modulus $B_{0}$ (GPa)	−0.5	1.03	1.21	3.13
shear modulus $G_{V}$ (GPa)	−0.39	0.57	1.356	1.478
Young’s modulus *Y* (GPa)	−0.928	1.448	2.96	3.83
Poisson’s ratio ν (GPa)	0.19	0.265	0.09	0.296
Lamé constants λ (GPa)	−0.24	0.646	0.298	2.14
Lamé constants μ (GPa)	−0.39	0.57	1.357	1.477
Kleinman parameter ξ (GPa)	0.134	0.05	−0.1	0.38
Shear constant $C^{\prime }$ (GPa)	−0.776	2	1.54	2.45
Cauchy’s pressure $C^{\prime \prime }$ (GPa)	0.144	0.08	−1.77	0.67

**Table 6:** Elastic constants (clamped ion) $C_{11}$, $C_{12}$, $C_{13}$, $C_{33}$, $C_{44}$, and $C_{66}$ in units (×10^2^) GPa, for Si[111] NWs, Si[111]-P NWs and Si[111]-B NWs. a, b, and c denote the substitution of dopant at three different positions on SiNWs[111] structures.

	Si[111]	Si[111]-P			Si[111]-B		
		a	b	c	a	b	c
$C_{11}$	0.1572196	0.1009792	0.1059315	0.1086349	0.1071444	0.1086057	0.1065181
$C_{12}$	0.0264517	0.0318655	0.0306969	0.0278214	0.0224188	0.0241336	0.0265312
$C_{13}$	0.0353120	0.0287954	0.0273921	0.0302353	0.0321996	0.0289576	0.0296984
$C_{33}$	0.1901712	0.2155796	0.2175706	0.2318382	0.2118774	0.2106602	0.2293764
$C_{44}$	0.0398118	0.0325173	0.0306316	0.0374749	0.0404230	0.0398787	0.0400541
$C_{66}$	0.0314324	0.0144414	0.0164137	0.0163026	0.0146337	0.0125096	0.0175745

**Table 7:** Bulk modulus ($B_{0}$), shear modulus ($G_{V}$), Young’s modulus (*Y*), Poisson’s ratio (ν), Lamé constants (λ), Lamé constants (μ), Kleinman parameter (ξ), Shear constant ($C^{\prime }$) and Cauchy’s pressure ($C^{\prime \prime }$) in lateral axis (lat.) for Si[001] NWs, Si[001]-B NWs and Si[001]-P NWs.

	Si[001]	Si[001]-B	Si[001]-P
	Lat.	Lat.	Lat.
Bulk modulus $B_{0}$ (GPa)	1.21	6	5.68
shear modulus $G_{V}$ (GPa)	1.356	4.572	4.59
Young’s modulus *Y* (GPa)	2.96	10.93	10.85
Poisson’s ratio ν (GPa)	0.09	0.196	0.18
Lamé constants λ (GPa)	0.298	2.94	2.586
Lamé constants μ (GPa)	1.357	4.57	4.59
Kleinman parameter ξ (GPa)	−0.1	0.578	0.57
Shear constant $C^{\prime }$ (GPa)	1.54	2.64	2.57
Cauchy’s pressure $C^{\prime \prime }$ (GPa)	−1.77	−1.62	−1.97

**Table 8:** Elastic moduli $B_{0}$, $G_{V}$, *Y*, ν, λ, μ, ξ, $C^{\prime }$ and $C^{\prime \prime }$ in longitudinal axis (long.) for Si[001] NWs, Si[001]-B NWs and Si[001]-P NWs.

	Si[001]	Si[001]-B	Si[001]-P
	Long.	Long.	Long.
Bulk modulus $B_{0}$ (GPa)	3.13	9.9	8.61
shear modulus $G_{V}$ (GPa)	1.478	5.83	5.34
Young’s modulus *Y* (GPa)	3.83	14.62	13.27
Poisson’s ratio ν (GPa)	0.296	0.25	0.24
Lamé constants λ (GPa)	2.14	5.9	4.94
Lamé constants μ (GPa)	1.477	5.84	5.35
Kleinman parameter ξ (GPa)	0.38	0.365	0.405
Shear constant $C^{\prime }$ (GPa)	2.45	8.17	6.36
Cauchy’s pressure $C^{\prime \prime }$ (GPa)	0.67	0.19	−0.29

**Table 9:** Bulk modulus ($B_{0}$), shear modulus ($G_{V}$), Young’s modulus (*Y*), Poisson’s ratio (ν), Lamé constants (λ), Lamé constants (μ), Kleinman parameter (ξ), Shear constant ($C^{\prime }$) and Cauchy’s pressure ($C^{\prime \prime }$) in both lateral axis (lat.) and longitudinal axis (long.) for Si[111] NWs, Si[111]-B NWs and Si[111]-P NWs. a, b, and c denote the substitution of dopant at three different positions on SiNWs[111] structures.

	Si[111]		Si[111]-B						Si[111]-P					
			a		b		c		a		b		c	
	Lat.	Long.	Lat.	Long.	Lat.	Long.	Lat.	Long.	Lat.	Long.	Lat.	Long.	Lat.	Long.
Bulk modulus $B_{0}$ (GPa)	7	8.68	5.06	9.2	5.2	8.92	5.31	9.62	5.45	9.1	5.53	9.07	5.47	9.74
shear modulus $G_{V}$ (GPa)	5	4.98	4.09	4.46	4.09	4.37	4	5.04	3.314	4.6	3.32	4.78	3.86	5
Young’s Modulus *Y* (GPa)	12.11	12.5	9.66	11.5	9.72	11.27	9.6	12.87	8.26	11.8	8.3	12.2	9.37	12.8
Poisson’s ratio ν (GPa)	0.21	0.26	0.18	0.29	0.188	0.29	0.2	0.277	0.247	0.28	0.25	0.27	0.21	0.28
Lamé constants λ (GPa)	3.62	5.37	2.3	6.15	2.46	6.03	2.66	6.26	3.23	5.86	3.32	5.63	2.8	6.36
Lamé constants μ (GPa)	5	4.96	4.09	4.457	4.09	4.378	4	5.04	3.312	4.6	3.32	4.8	3.87	5
Kleinman parameter ξ (GPa)	0.32	0.33	0.36	0.3	0.37	0.288	0.4	0.28	0.46	0.284	0.43	0.276	0.4	0.28
Shear constant $C^{\prime }$ (GPa)	6.54	7.73	4.23	8.98	4.22	9.05	4	9.98	3.41	9.33	3.8	9.5	4.04	10.07
Cauchy’s pressure $C^{\prime }$ (GPa)	−1.34	0.39	−1.76	1.76	−1.59	1.64	−1.35	1.22	−0.07	1.44	0.006	1.1	−0.96	1.39

## Data Availability

All data from this study are available from the corresponding author upon request.
